# 3,4,6-Tri-*O*-acetyl-1,2-[(*S*)-ethyl­idene]-β-d-mannopyran­ose

**DOI:** 10.1107/S1600536812032369

**Published:** 2012-07-21

**Authors:** Henok H. Kinfe, Felix L. Makolo, Zanele Phasha, Alfred Muller

**Affiliations:** a Research Center for Synthesis and Catalysis, Department of Chemistry, University of Johannesburg (APK Campus), PO Box 524, Auckland Park, Johannesburg, 2006, South Africa

## Abstract

In the title compound, C_14_H_20_O_9_, the six-membered pyran and the five-membered dioxalane rings adopt chair and twisted conformations, respectively. In the crystal, the mol­ecules are linked by C—H⋯O inter­actions.

## Related literature

For orthogonal protection in carbohydrate chemistry, see: Wuts & Greene (2007 ▶); Betaneli *et al.* (1982 ▶). For background to the synthetic methodology, see: Doores *et al.* (2010 ▶). For ring puckering analysis, see: Cremer & Pople (1975 ▶). 
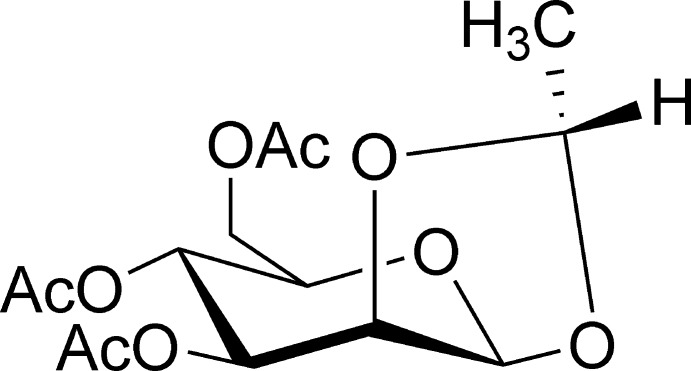


## Experimental

### 

#### Crystal data

C_14_H_20_O_9_
*M* *_r_* = 332.3
Orthorhombic, 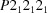

*a* = 7.0494 (3) Å
*b* = 14.6994 (7) Å
*c* = 15.3608 (7) Å
*V* = 1591.72 (12) Å^3^
*Z* = 4
Cu *K*α radiation
μ = 1.01 mm^−1^
*T* = 100 K
0.16 × 0.16 × 0.12 mm

#### Data collection

Bruker APEX DUO 4K-CCD diffractometer
Absorption correction: multi-scan (*SADABS*; Bruker, 2008 ▶) *T*_min_ = 0.856, *T*_max_ = 0.889
23454 measured reflections
2701 independent reflections
2684 reflections with *I* > 2σ(*I*)
*R*_int_ = 0.028

#### Refinement

*R*[*F* ^2^ > 2σ(*F* ^2^)] = 0.026
*wR*(*F* ^2^) = 0.085
*S* = 1.20
2701 reflections
212 parameters
H-atom parameters constrained
Δρ_max_ = 0.26 e Å^−3^
Δρ_min_ = −0.31 e Å^−3^
Absolute structure: Flack (1983 ▶), 1110 Friedel Pairs
Flack parameter: 0.06 (15)

### 

Data collection: *APEX2* (Bruker, 2011 ▶); cell refinement: *SAINT* (Bruker, 2008 ▶); data reduction: *SAINT* and *XPREP* (Bruker, 2008 ▶); program(s) used to solve structure: *SHELXS97* (Sheldrick, 2008 ▶); program(s) used to refine structure: *SHELXL97* (Sheldrick, 2008 ▶); molecular graphics: *DIAMOND* (Brandenburg & Putz, 2005 ▶); software used to prepare material for publication: *WinGX* (Farrugia, 1999 ▶).

## Supplementary Material

Crystal structure: contains datablock(s) global, I. DOI: 10.1107/S1600536812032369/xu5592sup1.cif

Structure factors: contains datablock(s) I. DOI: 10.1107/S1600536812032369/xu5592Isup2.hkl

Additional supplementary materials:  crystallographic information; 3D view; checkCIF report

## Figures and Tables

**Table 1:** Hydrogen-bond geometry (Å, °)

*D*—H⋯*A*	*D*—H	H⋯*A*	*D*⋯*A*	*D*—H⋯*A*
C1—H1*C*⋯O3^i^	0.98	2.55	3.511 (2)	167
C8—H8⋯O7^i^	1.00	2.49	3.317 (2)	140
C12—H12*A*⋯O4^ii^	0.98	2.56	3.469 (2)	154
C12—H12*B*⋯O1^iii^	0.98	2.51	3.460 (2)	163
C14—H14*B*⋯O9^iv^	0.98	2.53	3.361 (2)	142

Symmetry codes: (i) 
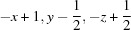
; (ii) 
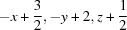
; (iii) 
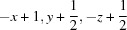
; (iv) 
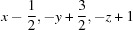
.

**Fig. 1.**
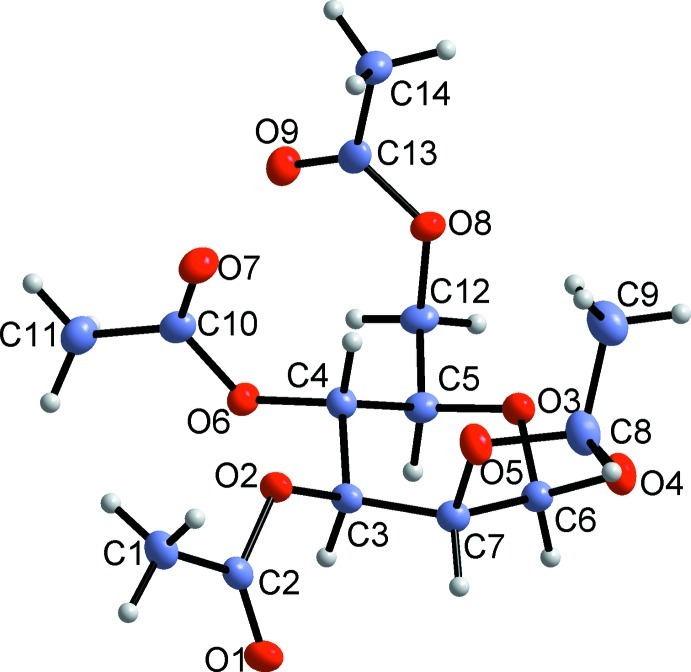
A view of (1). Displacement ellipsoids are drawn at the 50% probability level.

## supplementary crystallographic information

### Comment

Ethylidene acetals are important functional groups for orthogonal protection in carbohydrate chemistry (Wuts & Greene, 2007; Betaneli *et al.*, 1982). The title compound is a key intermediate for the preparation of polysaccharides which exhibit strong activity against the HIV-1 virus (Doores *et al.*, 2010). Herein, we report the crystal structure of 3,4,6-*tri*-*O*-acetyl-1,2-*O*-[*S*-ethylidene] -β-*D*-mannopyranoside to confirm its absolute configuration.

The title compound C_14_H_20_O_9_ (see Fig. 1, and Scheme 1) crystallizes in the *P*2_1_2_1_2_1_ (*Z* = 4) space group. Puckering analysis confirms the twisted conformation of the five membered dioxalane ring, with puckering parameter values of q_2_ = 0.347 (3) Å, and φ_2_ = 61.9 (4)°; and that of the six membered pyran chair conformation ring as q_2_ = 0.163 (3) Å, q_3_ = -0.526 (3) Å, Q = 0.550 (3) Å and φ_2_ = 253.3 (9)° (see Cremer & Pople, 1975). The dioxalane ring is twisted on C6–C7.

The molecules are linked by C-H···O interactions (see Table 1).

### Experimental

A solution of 2,3,4,6-*tetra*-*O*-acetyl -α-*D*-mannopyranosyl bromide (150 mg, 0.36 mmol) in acetonitrile (3 ml) was treated with sodium boron hydride (250 mg, 6.61 mmol) and the reaction mixture was stirred at room temperature for 12 h. The mixture was then diluted with chloroform and washed with water three times. The organic layer was dried over anhydrous Na_2_SO_4_, filtered and evaporated *in vacuo* to give an oil. The oily residue was crashed with methanol to afford 70% of the target compound as white crystals.

Analytical data: mp: 108-110 °C (Lit. 113-115 °C; Betaneli *et al.*, 1982); ^1^H NMR (CDCl_3_, 400 MHz): δ 5.40-5.10 (m, 4H), 4.31-4.03 (m, 3H), 3.72-3.64 (m, 1H), 2.09 (s, 3H), 2.04 (s, 3H), 2.02 (s, 3H), 1.51 (d, *J* = 4.8 Hz, 3H); ^13^C NMR (CDCl_3_, 400 MHz): δ 170.7, 170.3, 169.5, 104.8, 96.5, 71.6, 70.6, 66.0, 62.5, 21.6, 20.7, 20.7.

### Refinement

All hydrogen atoms were positioned in geometrically idealized positions with C–H = 1.00 Å (methine), 0.99 Å (methylene), and 0.98 Å (methyl). All hydrogen atoms were allowed to ride on their parent atoms with *U*_iso_(H) = 1.2*U*_eq_, except for the methyl where *U*_iso_(H) = 1.5*U*_eq_ was utilized. The initial positions of methyl hydrogen atoms were located from a Fourier difference map and refined as a fixed rotor. The D enantiomer refined to a final Flack parameter of 0.06 (15). The highest residual electron density of 0.50 e.Å^-3^ is 0.93 Å from H14B.

### Crystal data

**Table:**

C_14_H_20_O_9_	*F*(000) = 704
*M**_r_* = 332.3	*D*_x_ = 1.387 Mg m^−^^3^
Orthorhombic, *P*2_1_2_1_2_1_	Cu *K*α radiation, λ = 1.54178 Å
Hall symbol: P 2ac 2ab	Cell parameters from 9727 reflections
*a* = 7.0494 (3) Å	θ = 6.5–65.8°
*b* = 14.6994 (7) Å	µ = 1.01 mm^−^^1^
*c* = 15.3608 (7) Å	*T* = 100 K
*V* = 1591.72 (12) Å^3^	Cube, colourless
*Z* = 4	0.16 × 0.16 × 0.12 mm

### Data collection

**Table:**

Bruker APEX DUO 4K-CCD diffractometer	2701 independent reflections
Radiation source: Incoatec IµS microfocus X-ray source	2684 reflections with *I* > 2σ(*I*)
Incoatec Quazar Multilayer Mirror monochromator	*R*_int_ = 0.028
Detector resolution: 8.4 pixels mm^-1^	θ_max_ = 66.2°, θ_min_ = 4.2°
φ and ω scans	*h* = −8→8
Absorption correction: multi-scan (*SADABS*; Bruker, 2008)	*k* = −16→14
*T*_min_ = 0.856, *T*_max_ = 0.889	*l* = −18→17
23454 measured reflections	

### Refinement

**Table:**

Refinement on *F*^2^	Secondary atom site location: difference Fourier map
Least-squares matrix: full	Hydrogen site location: inferred from neighbouring sites
*R*[*F*^2^ > 2σ(*F*^2^)] = 0.026	H-atom parameters constrained
*wR*(*F*^2^) = 0.085	*w* = 1/[σ^2^(*F*_o_^2^) + (0.0494*P*)^2^ + 0.3436*P*] where *P* = (*F*_o_^2^ + 2*F*_c_^2^)/3
*S* = 1.20	(Δ/σ)_max_ = 0.001
2701 reflections	Δρ_max_ = 0.26 e Å^−^^3^
212 parameters	Δρ_min_ = −0.31 e Å^−^^3^
0 restraints	Absolute structure: Flack (1983), 1110 Friedel Pairs
Primary atom site location: structure-invariant direct methods	Flack parameter: 0.06 (15)

### Special details

**Table:**

Experimental. The intensity data was collected on a Bruker Apex DUO 4 K CCD diffractometer using an exposure time of 5 s/frame. A total of 4548 frames were collected with a frame width of 1° covering up to θ = 66.21° with 97.7% completeness accomplished.
Geometry. All s.u.'s (except the s.u. in the dihedral angle between two l.s. planes) are estimated using the full covariance matrix. The cell s.u.'s are taken into account individually in the estimation of s.u.'s in distances, angles and torsion angles; correlations between s.u.'s in cell parameters are only used when they are defined by crystal symmetry. An approximate (isotropic) treatment of cell s.u.'s is used for estimating s.u.'s involving l.s. planes.
Refinement. Refinement of *F*^2^ against ALL reflections. The weighted *R*-factor *wR* and goodness of fit *S* are based on *F*^2^, conventional *R*-factors *R* are based on *F*, with *F* set to zero for negative *F*^2^. The threshold expression of *F*^2^ > 2σ(*F*^2^) is used only for calculating *R*-factors(gt) *etc*. and is not relevant to the choice of reflections for refinement. *R*-factors based on *F*^2^ are statistically about twice as large as those based on *F*, and *R*- factors based on ALL data will be even larger.

### Fractional atomic coordinates and isotropic or equivalent isotropic displacement parameters (Å^2^)

**Table:**

	*x*	*y*	*z*	*U*_iso_*/*U*_eq_	
C1	0.1916 (3)	0.55884 (11)	0.36854 (11)	0.0238 (4)	
H1A	0.0905	0.5131	0.3687	0.036*	
H1B	0.2086	0.5828	0.4276	0.036*	
H1C	0.3102	0.5309	0.3487	0.036*	
C2	0.1392 (2)	0.63445 (10)	0.30873 (10)	0.0191 (3)	
C3	0.2253 (2)	0.78379 (10)	0.26420 (10)	0.0182 (3)	
H3	0.085	0.7938	0.2623	0.022*	
C4	0.3175 (2)	0.86302 (10)	0.30997 (10)	0.0181 (3)	
H4	0.4551	0.8504	0.3202	0.022*	
C5	0.2937 (2)	0.94827 (11)	0.25454 (10)	0.0199 (3)	
H5	0.156	0.9571	0.2413	0.024*	
C6	0.3204 (2)	0.86280 (11)	0.12195 (10)	0.0200 (3)	
H6	0.1988	0.8811	0.0933	0.024*	
C7	0.2989 (2)	0.77323 (10)	0.17178 (10)	0.0195 (3)	
H7	0.2167	0.7302	0.1384	0.023*	
C8	0.5782 (3)	0.76982 (12)	0.09524 (12)	0.0275 (4)	
H8	0.5843	0.7181	0.053	0.033*	
C9	0.7739 (3)	0.80202 (14)	0.11606 (13)	0.0346 (4)	
H9A	0.7676	0.8491	0.1611	0.052*	
H9B	0.8325	0.8273	0.0635	0.052*	
H9C	0.85	0.7508	0.1372	0.052*	
C10	0.3704 (2)	1.03347 (11)	0.29613 (11)	0.0214 (4)	
H10A	0.3591	1.0852	0.2552	0.026*	
H10B	0.2967	1.048	0.3492	0.026*	
C11	0.6193 (2)	1.03193 (10)	0.40213 (11)	0.0203 (4)	
C12	0.8252 (3)	1.01153 (13)	0.41372 (11)	0.0258 (4)	
H12A	0.8696	1.038	0.4686	0.039*	
H12B	0.8971	1.0378	0.3652	0.039*	
H12C	0.8441	0.9455	0.4149	0.039*	
C13	0.3177 (2)	0.85697 (10)	0.46588 (11)	0.0208 (3)	
C14	0.1957 (3)	0.87231 (12)	0.54383 (11)	0.0274 (4)	
H14A	0.267	0.857	0.5966	0.041*	
H14B	0.0827	0.8337	0.54	0.041*	
H14C	0.1573	0.9363	0.5461	0.041*	
O1	0.00432 (18)	0.63631 (8)	0.26050 (8)	0.0251 (3)	
O2	0.26442 (16)	0.70374 (7)	0.31509 (7)	0.0192 (2)	
O3	0.39448 (16)	0.93390 (8)	0.17474 (8)	0.0213 (3)	
O4	0.46022 (18)	0.84164 (8)	0.06055 (8)	0.0251 (3)	
O5	0.48923 (17)	0.74080 (7)	0.17374 (8)	0.0234 (3)	
O6	0.56740 (16)	1.01897 (7)	0.31844 (7)	0.0202 (3)	
O7	0.51158 (18)	1.05559 (8)	0.45868 (8)	0.0249 (3)	
O8	0.22067 (16)	0.87679 (7)	0.39160 (7)	0.0201 (3)	
O9	0.47936 (18)	0.83119 (8)	0.46649 (8)	0.0265 (3)	

### Atomic displacement parameters (Å^2^)

**Table:**

	*U*^11^	*U*^22^	*U*^33^	*U*^12^	*U*^13^	*U*^23^
C1	0.0263 (9)	0.0187 (8)	0.0263 (8)	−0.0010 (7)	0.0026 (7)	0.0042 (7)
C2	0.0200 (8)	0.0172 (8)	0.0200 (8)	−0.0019 (6)	0.0034 (7)	−0.0019 (6)
C3	0.0178 (7)	0.0158 (7)	0.0210 (7)	−0.0010 (6)	−0.0022 (6)	0.0033 (6)
C4	0.0179 (7)	0.0177 (8)	0.0186 (7)	0.0006 (6)	0.0005 (6)	0.0012 (6)
C5	0.0192 (7)	0.0181 (8)	0.0224 (8)	0.0014 (6)	−0.0036 (6)	0.0007 (6)
C6	0.0210 (8)	0.0197 (8)	0.0192 (8)	−0.0022 (6)	−0.0015 (6)	0.0008 (6)
C7	0.0197 (8)	0.0170 (7)	0.0216 (7)	0.0000 (6)	−0.0008 (7)	0.0005 (6)
C8	0.0314 (9)	0.0222 (8)	0.0290 (9)	0.0041 (7)	0.0065 (8)	0.0050 (7)
C9	0.0309 (10)	0.0329 (10)	0.0399 (10)	0.0006 (8)	0.0032 (8)	0.0091 (8)
C10	0.0198 (8)	0.0186 (8)	0.0259 (8)	0.0014 (6)	−0.0065 (7)	0.0008 (7)
C11	0.0275 (9)	0.0128 (7)	0.0207 (8)	−0.0017 (7)	−0.0031 (7)	0.0031 (7)
C12	0.0243 (9)	0.0293 (9)	0.0238 (8)	−0.0009 (7)	−0.0044 (7)	0.0016 (7)
C13	0.0247 (9)	0.0147 (8)	0.0229 (8)	−0.0006 (6)	−0.0043 (7)	−0.0007 (6)
C14	0.0315 (9)	0.0283 (9)	0.0222 (8)	0.0003 (8)	−0.0005 (7)	−0.0040 (7)
O1	0.0234 (6)	0.0225 (6)	0.0294 (6)	−0.0049 (5)	−0.0047 (5)	0.0021 (5)
O2	0.0208 (6)	0.0158 (5)	0.0210 (5)	−0.0030 (4)	−0.0030 (4)	0.0039 (4)
O3	0.0246 (6)	0.0186 (5)	0.0208 (5)	−0.0046 (5)	−0.0017 (5)	0.0019 (5)
O4	0.0271 (6)	0.0229 (6)	0.0254 (6)	0.0029 (5)	0.0053 (5)	0.0057 (5)
O5	0.0241 (6)	0.0202 (5)	0.0259 (6)	0.0051 (5)	0.0051 (5)	0.0060 (5)
O6	0.0199 (6)	0.0203 (5)	0.0204 (5)	−0.0003 (4)	−0.0039 (5)	−0.0014 (5)
O7	0.0286 (6)	0.0242 (6)	0.0219 (6)	0.0031 (5)	0.0011 (5)	−0.0005 (5)
O8	0.0200 (6)	0.0218 (5)	0.0186 (5)	0.0022 (5)	−0.0007 (5)	−0.0005 (4)
O9	0.0257 (7)	0.0287 (6)	0.0250 (6)	0.0050 (5)	−0.0056 (5)	−0.0003 (5)

### Geometric parameters (Å, º)

**Table:**

C1—C2	1.489 (2)	C8—O5	1.425 (2)
C1—H1A	0.98	C8—O4	1.446 (2)
C1—H1B	0.98	C8—C9	1.493 (3)
C1—H1C	0.98	C8—H8	1
C2—O1	1.206 (2)	C9—H9A	0.98
C2—O2	1.3514 (19)	C9—H9B	0.98
C3—O2	1.4394 (18)	C9—H9C	0.98
C3—C4	1.508 (2)	C10—O6	1.4461 (19)
C3—C7	1.520 (2)	C10—H10A	0.99
C3—H3	1	C10—H10B	0.99
C4—O8	1.4420 (19)	C11—O7	1.205 (2)
C4—C5	1.524 (2)	C11—O6	1.350 (2)
C4—H4	1	C11—C12	1.493 (2)
C5—O3	1.433 (2)	C12—H12A	0.98
C5—C10	1.506 (2)	C12—H12B	0.98
C5—H5	1	C12—H12C	0.98
C6—O4	1.399 (2)	C13—O9	1.201 (2)
C6—O3	1.422 (2)	C13—O8	1.3620 (19)
C6—C7	1.530 (2)	C13—C14	1.492 (2)
C6—H6	1	C14—H14A	0.98
C7—O5	1.424 (2)	C14—H14B	0.98
C7—H7	1	C14—H14C	0.98
			
C2—C1—H1A	109.5	O4—C8—C9	112.28 (15)
C2—C1—H1B	109.5	O5—C8—H8	109.9
H1A—C1—H1B	109.5	O4—C8—H8	109.9
C2—C1—H1C	109.5	C9—C8—H8	109.9
H1A—C1—H1C	109.5	C8—C9—H9A	109.5
H1B—C1—H1C	109.5	C8—C9—H9B	109.5
O1—C2—O2	122.85 (14)	H9A—C9—H9B	109.5
O1—C2—C1	126.29 (15)	C8—C9—H9C	109.5
O2—C2—C1	110.86 (13)	H9A—C9—H9C	109.5
O2—C3—C4	107.19 (12)	H9B—C9—H9C	109.5
O2—C3—C7	110.99 (12)	O6—C10—C5	108.84 (13)
C4—C3—C7	111.54 (13)	O6—C10—H10A	109.9
O2—C3—H3	109	C5—C10—H10A	109.9
C4—C3—H3	109	O6—C10—H10B	109.9
C7—C3—H3	109	C5—C10—H10B	109.9
O8—C4—C3	108.04 (12)	H10A—C10—H10B	108.3
O8—C4—C5	108.55 (12)	O7—C11—O6	123.81 (15)
C3—C4—C5	109.08 (12)	O7—C11—C12	125.78 (16)
O8—C4—H4	110.4	O6—C11—C12	110.40 (14)
C3—C4—H4	110.4	C11—C12—H12A	109.5
C5—C4—H4	110.4	C11—C12—H12B	109.5
O3—C5—C10	107.89 (13)	H12A—C12—H12B	109.5
O3—C5—C4	107.58 (12)	C11—C12—H12C	109.5
C10—C5—C4	114.01 (13)	H12A—C12—H12C	109.5
O3—C5—H5	109.1	H12B—C12—H12C	109.5
C10—C5—H5	109.1	O9—C13—O8	123.42 (15)
C4—C5—H5	109.1	O9—C13—C14	126.07 (16)
O4—C6—O3	106.79 (13)	O8—C13—C14	110.51 (14)
O4—C6—C7	102.44 (12)	C13—C14—H14A	109.5
O3—C6—C7	112.54 (12)	C13—C14—H14B	109.5
O4—C6—H6	111.5	H14A—C14—H14B	109.5
O3—C6—H6	111.5	C13—C14—H14C	109.5
C7—C6—H6	111.5	H14A—C14—H14C	109.5
O5—C7—C3	109.66 (13)	H14B—C14—H14C	109.5
O5—C7—C6	101.86 (12)	C2—O2—C3	116.85 (12)
C3—C7—C6	114.39 (13)	C6—O3—C5	114.45 (12)
O5—C7—H7	110.2	C6—O4—C8	108.62 (12)
C3—C7—H7	110.2	C7—O5—C8	107.27 (12)
C6—C7—H7	110.2	C11—O6—C10	117.71 (13)
O5—C8—O4	106.08 (13)	C13—O8—C4	117.42 (12)
O5—C8—C9	108.69 (15)		

### Hydrogen-bond geometry (Å, º)

**Table:**

*D*—H···*A*	*D*—H	H···*A*	*D*···*A*	*D*—H···*A*
C1—H1*C*···O3^i^	0.98	2.55	3.511 (2)	167
C8—H8···O7^i^	1.00	2.49	3.317 (2)	140
C12—H12*A*···O4^ii^	0.98	2.56	3.469 (2)	154
C12—H12*B*···O1^iii^	0.98	2.51	3.460 (2)	163
C14—H14*B*···O9^iv^	0.98	2.53	3.361 (2)	142

Symmetry codes: (i) −*x*+1, *y*−1/2, −*z*+1/2; (ii) −*x*+3/2, −*y*+2, *z*+1/2; (iii) −*x*+1, *y*+1/2, −*z*+1/2; (iv) *x*−1/2, −*y*+3/2, −*z*+1.
